# Effects of In-Hospital Physical Therapy on Activities of Daily Living in Patients with Hepatocellular Carcinoma

**DOI:** 10.3390/ijerph17239098

**Published:** 2020-12-06

**Authors:** Hayato Narao, Keisuke Hirota, Shunji Koya, Manabu Tomita, Yuta Manako, Satosi Ogawa, Naomi Nakao, Tsubasa Tsutsumi, Dan Nakano, Ryuki Hashida, Takumi Kawaguchi, Hiroo Matsuse, Hiroaki Nagamatu, Takuji Torimura

**Affiliations:** 1 Department of Rehabilitation, Yame General Hospital, Yame 834-0034, Japan; hayatonarao198071@gmail.com (H.N.); ymhp1892@yamehp.jp (Y.M.); 2 Division of Rehabilitation, Kurume University Hospital, Kurume 830-0011, Japan; hirota_keisuke@kurume-u.ac.jp (K.H.); kouya_shunji@kurume-u.ac.jp (S.K.); hashida_ryuuki@med.kurume-u.ac.jp (R.H.); matsuse_hiroh@kurume-u.ac.jp (H.M.); 3 Department of Rehabilitation, Saga Central Hospital, Saga 849-8522, Japan; sp4u7m99@ray.ocn.ne.jp; 4 Department of Nursing, Yame General Hospital, Yame 834-0034, Japan; ymhp0311@yamehp.jp (S.O.); ymhp1430@yamehp.jp (N.N.); 5 Division of Gastroenterology, Department of Medicine, Kurume University School of Medicine, Kurume 830-0011, Japan; tsutsumi_tsubasa@med.kurume-u.ac.jp (T.T.); nakano_dan@med.kurume-u.ac.jp (D.N.); tori@med.kurume-u.ac.jp (T.T.); 6 Department of Orthopedics, Kurume University School of Medicine, Kurume 830-0011, Japan; 7 Department of Gastroenterology, Juntendo University, Tokyo 113-8421, Japan; angio0112tiger@gmail.com

**Keywords:** supervised physiotherapy, functional independence measure, hepatoma, liver, hospitalization

## Abstract

Activities of daily living (ADL) are frequently impaired in patients with hepatocellular carcinoma (HCC). In this retrospective study, we aimed to investigate the effects of physical therapy on ADLs in patients with HCC during hospitalization for cancer treatment. Nineteen patients with HCC were enrolled. During hospitalization, patients performed a combination of resistance training, stretching, and aerobic exercise (20–60 min/day). ADLs were assessed using the functional independence measure (FIM). Changes in FIM were evaluated by before–after analysis. No significant difference was seen in Child–Pugh class before and after physical therapy. The bilateral knee extension strength and chair stand test were significantly increased after physical therapy compared with before physical therapy (*p* = 0.001 and *p* = 0.008, respectively). The total FIM score was significantly increased after physical therapy compared with that before physical therapy (*p* = 0.0156). Among the 18 indexes of FIM, the stairs index was significantly improved after physical therapy compared with that before physical therapy (5.9 vs. 6.4 points, *p* = 0.0241). We demonstrated that physical therapy improved muscle strength without worsening liver function. Furthermore, physical therapy improved FIM, especially in the stairs index, in patients with HCC. Thus, physical therapy may be beneficial in patients with HCC during cancer treatment.

## 1. Introduction

Activities of daily living (ADLs) are frequently impaired in patients with cancer [1]. Impairment of ADLs is associated with a poor response to cancer treatment, mortality, and quality of life [1]. ADLs can be impaired by muscle atrophy and physical dysfunction [2]. In patients with hepatocellular carcinoma (HCC), sarcopenia is frequently seen. The muscle mass significantly decreases, and ADLs are impaired during hospitalization in patients with HCC [1,3,4]. One of the causative factors is that patients with HCC are generally instructed to be on bed rest during hospitalization [5].

The functional independence measure (FIM), reported by Granger et al. in 1983 [6], explores an individual’s physical, psychological, and social function, thus evaluating ADLs [7]. FIM consists of 18 physical/cognitive items, which are graded on a scale of 1–7 based on the level of independence [7]; a higher FIM score indicates more independence with ADLs [8]. The FIM is also used to evaluate the functional outcomes of medical rehabilitation [9]. FIM has been used to evaluate ADLs in patients with various cancers, including colorectal cancer [10]. FIM is associated with cognitive function, mortality, and quality of life in patients with various cancers [11,12,13].

Rehabilitation is effective at maintaining muscle mass, physical function, and cognitive function, resulting in an improvement of ADLs [13,14]. We developed a physical therapy program for patients with HCC, which is a combination of stretching, strength training, balance practice, and endurance training [15]. This physical therapy does not worsen liver function in patients with HCC [15]. In addition, physical therapy increased skeletal muscle mass in patients with HCC, even if they were receiving chemotherapy [14,16]. Physical therapy also improves cardiopulmonary function and muscle cramps during hospitalization for HCC treatment [15]. However, it remains unclear if physical therapy improves FIM scores in patients with HCC during hospitalization for cancer treatment.

In this study, we aimed to examine the impact of in-hospital physical therapy on FIM scores in patients with HCC.

## 2. Materials and Methods

### 2.1. Study Design

This is a retrospective chart review study to evaluate the impact of physical therapy on ADLs in patients with HCC during hospitalization for cancer treatment.

### 2.2. Ethics

The study protocol conformed to the ethical guidelines of the Declaration of Helsinki and was approved by the institutional review board of Public Yame General Hospital (approval #16-001). Informed consent from patients was obtained through an opt-out approach. Personal information was protected throughout the study. None of the patients were institutionalized.

Physical therapy for in-hospital patients with cancer is an approved healthcare service covered by the Ministry of Health, Labor, and Welfare of Japan’s health insurance. Therefore, a randomized controlled trial with an intentional setting of a non-physical therapy group is contrary to medical ethics.

### 2.3. Subjects

We enrolled 19 consecutive patients with HCC from January 2017 to September 2017. Inclusion criteria were hospitalized patients with HCC who (1) were 20 years of age or older, (2) had a performance status of grade 0 to 1 as defined by the Eastern Cooperative Oncology Group [17], (3) were hospitalized for more than 1 week and had more than 4 days of physical therapy, and (4) agreed to exercise. Exclusion criteria were as follows: (1) risk of HCC rupture, (2) grade 2–4 hepatic encephalopathy according to the West Haven Criteria [18], (3) risk of esophageal varices rupture, (4) heart failure, (5) respiratory failure, (6) fever of ≥38 °C, (7) leg vein thrombus, and (8) orthopedic disease within the past year resulting in the patient being unable to walk.

### 2.4. Protocol for Physical Therapy

This physical therapy for patients with HCC has not been used before in our facility. During hospitalization for HCC treatment, patients were treated with exercise (20–60 min/day), as instructed by physical therapists who were certified in the rehabilitation of patients with cancer. The exercise consisted of three types of training according to the guidelines of the American College of Sports Medicine (Table 1) [19]. The exercise was initiated the day after HCC treatment, as previously described [15]. Patients were instructed to continue the exercises after discharge. In our study, patients received only physical therapy, but not occupational therapy and speech–language–hearing therapy.

#### 2.4.1. Resistance Training

Patients underwent strength training for 10–20 min, targeting the bilateral quadriceps femoris muscles, gastrocnemius, and iliopsoas muscle. Bilateral quadriceps femoris muscle strength training was performed at 60% of the maximum voluntary contraction (MVC; moderate to high intensity) using a Combit CB-1 (Minato Medical Science Co., Ltd., Osaka, Japan) [20]. The quadriceps, gastrocnemius, and iliopsoas muscles were trained using the patient’s weight. One set consisted of 10 repetitions, and a maximum of four sets were performed [15,19].

#### 2.4.2. Stretching

Patients performed stretches for 3–5 min, targeting the quadriceps femoris muscles, the iliopsoas muscle, hip adductor muscles, and gastrocnemius muscles. Stretches were statically held for 20 s at the point of feeling tightness or slight discomfort [15,19].

#### 2.4.3. Aerobic Exercise

Patients practiced voluntary walking with a goal of 10–30 min, depending on their motor skills. The intensity of exercise was adjusted to maintain a subjective rating of perceived exertion of 11–13 points on the Borg scale [15,19].

### 2.5. Diagnosis, Tumor Node Metastasis (TNM) Staging, and Treatment of HCC

HCC was diagnosed using a tumor biopsy or a combination of tests for serum tumor markers and imaging procedures, such as ultrasonography, computed tomography (CT), magnetic resonance imaging, and/or angiography. The clinical stage of HCC was evaluated by TNM staging based on the Liver Cancer Study Group of Japan criteria [21]. Treatment for HCC was selected based on the evidence-based clinical practice guidelines for HCC of The Japan Society of Hepatology [22].

### 2.6. Measurement of Psoas Muscle Index (PMI) and Skeletal Muscle Index (SMI)

PMI and SMI were evaluated using CT images obtained before and after physical therapy. The CT images were taken for the assessment of HCC in the outpatient department. A standard landmark for measuring the psoas muscle mass and skeletal muscle mass was the lower border of the third lumbar vertebra (L3). We measured the psoas muscle mass and skeletal muscle mass by manual tracings on CT images using Image-J software (National Institutes of Health, Bethesda, Maryland, USA, version Windows 10), and their sum was calculated [23]. Skeletal muscle mass and psoas muscle mass were normalized by the square of the height, and the data were expressed as PMI and SMI [24].

### 2.7. Measurement of Muscle Strength

#### 2.7.1. Maximum Muscle Strength Assessment of Knee Extension Strength

The maximum knee extension strength was measured during isometric contraction using Combit CB-1 according to the manufacturer’s instructions. To prevent injury, several knee extension exercises were performed before the measurement. The measurement angle was set at 90° of the knee joint flexion. The measurement posture was set to start with the trunk and both thighs secured with a belt while sitting on the seat of Combit CB-1, and the lower leg of the measurement side was secured to the attachment. During the measurement of maximum knee extension strength, a mechanical voice played from the Combit CB-1 asking the patient to apply force in the direction of knee extension. In addition, the measurer also asked the patient to put more force in the direction of knee extension. Each side of the knee extension strength was measured once. The measured value was displayed as the maximum knee extension strength on the Combit CB-1 monitor.

#### 2.7.2. Grip Strength

An analog grip strength meter (Takei Machine Industry, Niigata, Japan) was used to measure grip strength. Left and right grip strength were measured in a standing position with the upper limbs hanging down to the side of the body. The test was completed twice each in both the left and right arms. A resting interval of at least 30 s was allowed between each measurement. The grip strength was defined as the average value of the higher grip strength of the left and right arms [25,26].

#### 2.7.3. 30-Second Chair–Stand Test (CS-30)

We employed the CS-30 to evaluate lower extremity muscle strength with a high retest reliability [27]. CS-30 was measured according to previous reports [28]. The patients were asked to sit on a chair that was 40 cm high. They were asked to position their legs shoulder width apart and to cross their arms in front of their chest as the starting posture. A single attempt was given before the measurement of CS-30. The test lasted 30 s. The subject was instructed to repeatedly stand and sit as many times as possible with their arms crossed.

### 2.8. Measurement of Walking Speed

Walking speed in meters per second (m/s) was measured by a 10-m walking test (10MWT). The 10MWT was modified from a previous report [29]. A stopwatch was used to measure the steady-state walking speed. To record the steady state walking speed during the 10MWT, 3 m acceleration and deceleration zones were added at the beginning and end of the course. Thus, the total walking distance was 16 m. The watch was started and stopped when the patient’s toe passed over the lines marking the start and end of the 10-m course, respectively [26]. We conducted the 10MWT twice, and the average walking speed was used for the analysis. Two subjects used walking aids.

### 2.9. Measurement of FIM

FIM is an assessment tool comprising 18 items, including eating, grooming, bathing, dressing the upper body, dressing the lower body, toileting, bladder management, bowel management, bed/chair/wheelchair, toilet, tub/shower, walk/wheelchair, stairs, comprehension, expression, social interaction, problem solving, and memory. Thirteen items belong to the motor subscale, and five items belong to the cognitive subscale, including comprehension, expression, social interaction, problem solving, and memory [7]. All of the items were scored as follows: 1 (total assist), 2 (maximal assist), 3 (moderate assist), 4 (minimal assist), 5 (supervision), 6 (modified independence), and 7 (complete independence). The FIM item scores were added together for a total score ranging between 18 and 126, or a total motor score ranging between 13 and 91 and a total cognitive score ranging between 5 and 35 [30]. These assessments were evaluated by physical therapists qualified in cancer rehabilitation with more than 13 years of experience.

### 2.10. Biochemical Tests

The following blood biochemical tests were performed: serum levels of aminotransferase (AST), alanine aminotransferase (ALT), lactate dehydrogenase (LD), alkaline phosphatase (ALP), gamma-glutamyl transpeptidase (GGT), albumin, total bilirubin, blood urea nitrogen (BUN), creatinine, estimated glomerular filtration rate (eGFR), sodium, and potassium, which were measured using a BM-6070 (JEOL Ltd., Tokyo, Japan). Hemoglobin A1c (HbA1c) was measured using HA8181 (ARKRAY Inc. Kyoto, Japan). The prothrombin activity was measured using a CS2100 (Sysmex, Kobe, Japan). Alpha-fetoprotein and des-γ-carboxy prothrombin were measured using the Lumipulse G1200 (Fujirevio, Tokyo, Japan). The platelet count was measured using a Sysmex XE-5000. Ammonia was measured using Pocket Chem BA (ARKRAY Inc).

### 2.11. Statistical Analysis

Data are expressed as median (interquartile range (IQR)), range, or number. Changes in variables between before and after physical therapy were evaluated using Wilcoxon signed-rank tests. The level of statistical significance was set at *p* < 0.05. These analyses were performed using JMP Pro^®^ 14 (SAS Institute Inc., Cary, NC, USA).

## 3. Results

### 3.1. Patient Characteristics

The patient characteristics are summarized in Table 2. The median age of the patients was 78 years, and 31.6% were women. The median BMI was 21.2 kg/m^2^. All of the patients had a performance status of grade 0 or 1, and the median FIM score was 126 points.

More than 80% of patients had stage II HCC. The median Child–Pugh score was 5, and 78.9% of patients were Child–Pugh class A. The prevalence of sarcopenia was 31.6%. The median hospitalization time was 10 days, and the median duration of exercise therapy was 5 days. The median right knee extension strength and left knee extension strength were 25.2 kgf and 24.2 kgf, respectively. The implementation rate was 66.7% for resistance training, stretching, standing stepping, calf raise, and half squat. The implementation rate for aerobic exercise was 75% (Table 2).

### 3.2. Changes in Liver and Renal Function after Physical Therapy

No significant differences were observed in the serum levels of AST, ALT, LD, ALP, and GGT (Table 3). There was no significant difference in the Child–Pugh score and Child–Pugh class before and after physical therapy (Table 3). No significant difference was seen in eGFR (Table 3).

#### 3.2.1. Changes in Skeletal Muscle Mass after Physical Therapy

**Figure 1:**
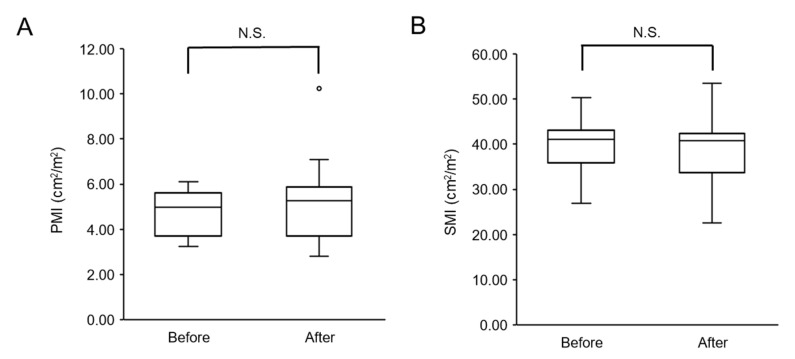
Changes in skeletal muscle mass after physical therapy: (**A**) psoas muscle index (PMI) and (**B**) skeletal muscle index (SMI). N.S.—not significant.

There was no significant decrease in PMI or SMI after physical therapy compared with before physical therapy (Figure 1A,B).

#### 3.2.2. Changes in Walking Speed and Muscle Strength after Physical Therapy

**Figure 2:**
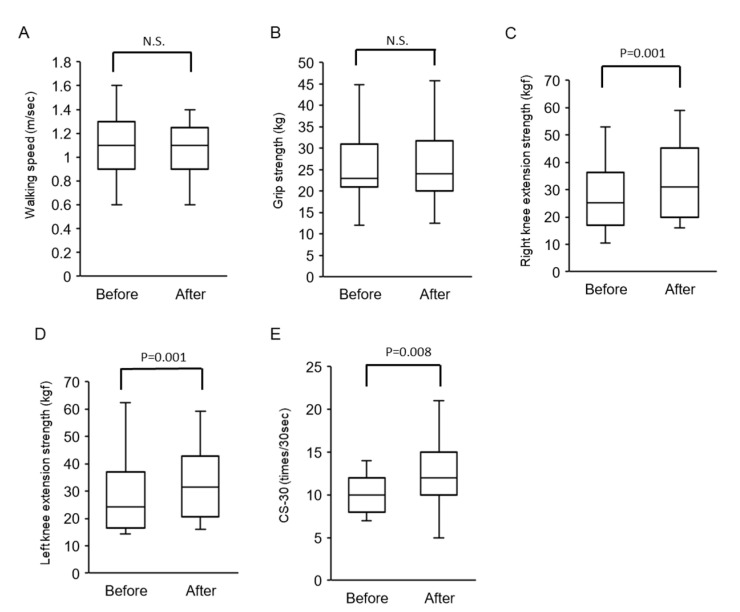
Changes in walking speed and muscle strength after physical therapy. (**A**) Walking speed, (**B**) grip strength, (**C**) right knee extension strength, (**D**) left knee extension strength, and (**E**) CS-30. Abbreviations: N.S.—not significant; CS-30—30-s chair–stand test.

No significant difference was seen in walking speed before and after physical therapy (Figure 2A). There was no significant difference in grip strength before and after physical therapy (Figure 2B). There was a significant increase in right and left knee extension strength after physical therapy compared with before physical therapy (Figure 2C,D). CS-30 was significantly improved after physical therapy compared with that before physical therapy (Figure 2E).

#### 3.2.3. Changes in FIM Score and All Items of FIM after Physical Therapy

**Figure 3:**
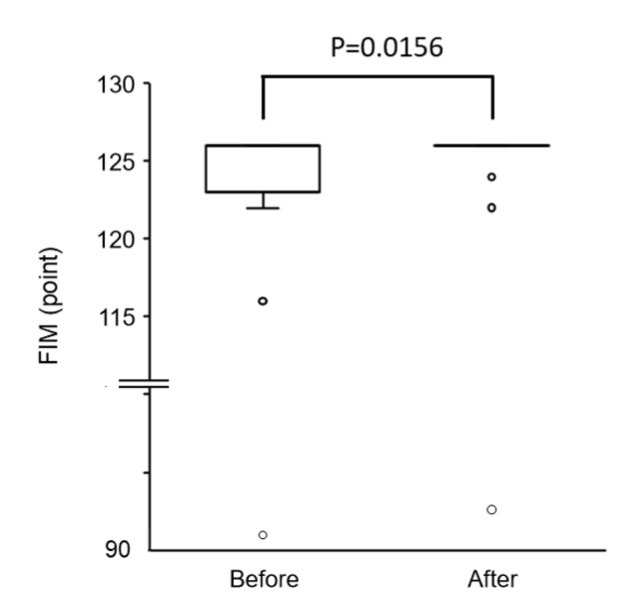
Changes in functional independence measure (FIM) score after physical therapy.

**Figure 4:**
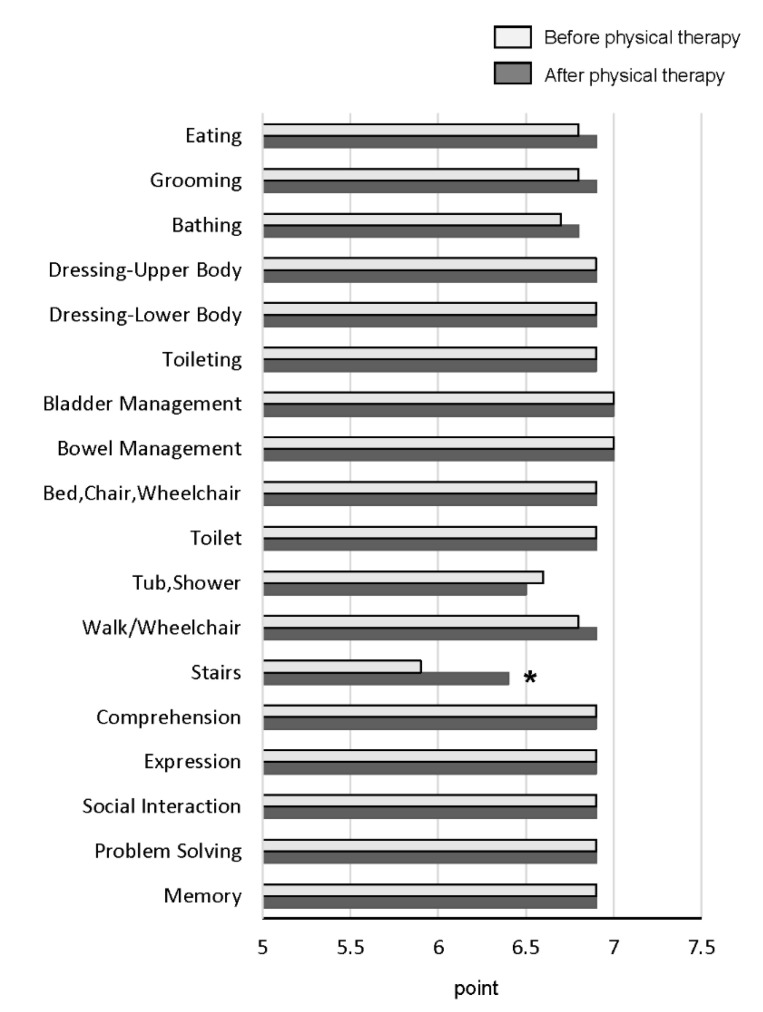
Changes in all items of FIM after physical therapy *, *p* < 0.01.

The total FIM score was significantly increased after physical therapy compared with before physical therapy (Figure 3). The stairs index is a motor index of FIM, and was the only item that was significantly improved after physical therapy (Figure 4). There were no significant changes in the scores of the other motor and cognitive indexes between before and after physical therapy (Figure 4).

## 4. Discussion

We demonstrated the safety and beneficial effects of in-hospital physical therapy, including resistance exercise, for patients with HCC. Liver and renal function did not worsen after physical therapy. Physical therapy with personalized intensity improved muscle strength with no reduction in muscle mass during hospitalization for HCC treatment. In addition, physical therapy improved FIM, especially in the stairs index, in patients with HCC during hospitalization for cancer treatment.

In our study, liver and renal function were not worsened by physical therapy. On the other hand, García-Pagàn et al. reported that moderate exercise increased the risk of variceal bleeding in patients with esophageal varices [31]. Saló et al. reported that moderate physical exercise may cause marked impairment in renal function in patients with liver cirrhosis [32]. Although it remains unclear why the liver function did not worsen with physical therapy, a possible reason is a difference in the type of exercises used in our study compared with that of previous studies. In our study, we mainly employed resistance training. However, previous studies employed aerobic exercise such as cycloergometric exercise [31,32]. The intensity of aerobic exercise is generally higher than that of resistance exercise [33], and cardiopulmonary function is known to be impaired in patients with HCC [34]. Therefore, aerobic exercise is a cardiopulmonary burden in patients with HCC. Our results may indicate that resistance exercise is more suitable than aerobic exercise for patients with HCC.

In this study, our physical therapy program was able to maintain muscle mass in patients with HCC. Our results were in good agreement with a previous report of HCC patients [14]. In addition, we demonstrated that our physical therapy program improved the muscle strength of the lower limbs. Although the reason the physical therapy program improved the muscle strength of the lower limbs remains unclear, it may be because resistance exercises were performed at a personalized intensity using a machine such as COMBIT CB-1. COMBIT CB-1 is able to precisely assess the maximal muscle strength of the lower limbs and allows the operator to set the appropriate intensity of exercise [20], which may result in increased muscle strength in the lower limbs. In fact, Nadia Schott et al. reported that exercise using machine training significantly increased the muscle strength of the lower limbs [35].

We first demonstrated that physical therapy increased the FIM score in patients with HCC. In previous studies, physical therapy has been reported to increase the FIM score in patients with various cancers, including colorectal cancer [10] and pancreatic cancer [36]. Thus, our results are in good agreement with these previous reports. Saotome et al. reported that an increase in FIM score by cancer rehabilitation was associated with prolonged survival in patients with cancer [37]. Moreover, an increase in FIM score through physical therapy has been reported to improve cognitive function in patients with cancer [13,38]. Furthermore, an increase in FIM score has been reported to be associated with an improved quality of life in patients with cancer. Therefore, evaluation of FIM seems to be important for the management of patients with HCC.

In this study, our physical therapy program increased the FIM score in patients with HCC. A possible reason for an increase in the FIM score is that we performed machine training with appropriate exercise intensity using COMBIT CB-1. Furthermore, a feature of our physical therapy program was that we mainly focused on exercising the lower-limb muscles, which is associated with physical performance [39]. As a result, physical therapy improved CS-30, an index for the strength of lower limb muscles in this study. In fact, among all 18 factors consisting of the FIM score, the ability to climb stairs was the only factor that was improved by physical therapy in this study. Matsufuji et al. also reported that chair stand exercises improved ADLs in patients on hemodialysis aged >60 years in a randomized controlled trial [40]. Yoshimura et al. also reported that an in-hospital chair–stand exercise was independently associated with an improvement in FIM-motor scores [41]. These findings indicate that physical therapy improved lower limb strength and subsequently the ability to climb stairs, leading to an increase in FIM score in this study.

This study has several limitations. First, this was a single-center study with a small sample size. Second, this was a retrospective chart review with no control group, which cannot prove the efficacy of physical therapy. Third, ADLs were assessed using only the FIM score and not by other methods such as the cancer functional assessment set [42]. Fourth, we could not perform a multivariate analysis because of the small sample size, and it remains unclear what type of physical therapy was associated with an improvement of FIM. Thus, the usefulness of physical therapy should be evaluated by a multi-center prospective study with a large sample size by using multiple assessments for ADLs. Fifth, Saotome et al. have reported that survival was significantly improved in patients with a total FIM score ≥80 after cancer rehabilitation [37]. In our study, all patients showed a total FIM score ≥ 80, even at the admission of the hospital. Therefore, it remains unclear if physical therapy had an impact on prognosis in our study. It also remains unclear which subscale of the FIM is important for survival. Thus, further studies should be focused on an association between an increase in FIM scores/the subscale of the FIM and an improvement in survival.

## 5. Conclusions

In this study, we performed in-hospital physical therapy, including resistance exercises, at a personalized intensity for patients with HCC. We demonstrated that in-hospital physical therapy did not worsen hepatic function. We also showed that exercise improved muscle strength with no reduction in muscle mass. Furthermore, we also showed that exercise improved the stair index of FIM in patients with HCC. Thus, in-hospital physical therapy may improve the ability to climb stairs in patients with HCC. Future research is needed to more clearly assess the benefits of physical therapy for ADL performance.

## Figures and Tables

**Table 1:** A protocol of physical therapy for patients with hepatocellular carcinoma (HCC).

Type of Exercise	Physical Therapy	Exercise Intensity	Frequency
1. Resistance training	Knee extension (Used Combit CB-1)	60% of MVC	2–3 days/week
	Standing stepping	Bodyweight	5 days/week
	Calf raise	Bodyweight	5 days/week
	Half squat	Bodyweight	5 days/week
2. Stretching	Lower limb stretching	Painless strength	3 days/week
3. Aerobic exercise	Walking	Borg scale 11–13 (Fairly light to somewhat hard)	Every day

**Table 2:** Patient characteristics.

	Reference Value	Median (IQR)	Range (Min–Max)
N	N/A	19	N/A
Age (years)	N/A	78.0 (73.0–82.0)	60–86
Sex (female/male)	N/A	6/13	N/A
Body mass index (kg/m^2^)	18.5–24.9	21.2 (20.7–26.5)	16.8–31.5
Performance status (0/1/2/3/4)	N/A	73.7%/26.3%/0%/0%/0% (14/5/0/0/0)	N/A
FIM score (points)	N/A	126 (123–126)	92–126
Walking assistance devise (yes/no)	N/A	10.5%/89.5% (2/17)	N/A
HCC stage (I/II/III/IV)	N/A	5.3%/84.1%/5.3%/5.3% (1/16/1/1)	N/A
HCV/HBV/others	N/A	78.9%/15.8%/5.3% (15/3/1)	N/A
Hospitalization (days)	N/A	10.0 (10.0–13.0)	7.0–28.0
Physical therapy (days)	N/A	5.0 (5.0–6.0)	4.0–15.0
Implementation rate of knee extension (%)	N/A	66.7 (51.8–77.5)	33.0–100.0
Implementation rate of standing stepping (%)	N/A	66.7 (51.8–77.5)	33.0–100.0
Implementation rate of calf raise (%)	N/A	66.7 (51.8–77.5)	33.0–100.0
Implementation rate of half squat (%)	N/A	66.7 (51.8–77.5)	33.0–100.0
Implementation rate of stretching (%)	N/A	66.7 (51.8–77.5)	33.0–100.0
Implementation rate of aerobic exercise (%)	N/A	75.0 (56.0–90.0)	0.0–100.0
Hepatic encephalopathy (Yes/No)	N/A	10.5%/89.5% (2/17)	N/A
Ascites (Yes/No)	N/A	10.5%/89.5% (2/17)	N/A
Walking speed (m/sec)	N/A	1.1 (0.9–1.3)	0.6–1.6
Six-minute walk test (m)	N/A	300 (262–340)	150.5–465.0
Grip strength (women/men) (kg)	N/A	17.1 (13.8–21.7) /28.0 (24.3–31.3)	12.0–22.3 /21.0–44.8
Grip strength (low/normal)	N/A	42.1%/57.9% (8/11)	N/A
Right knee extension strength (kgf)	N/A	25.2 (17.0–36.3)	10.5–53.0
Left knee extension strength (kgf)	N/A	24.2 (16.5–36.9)	14.3–62.3
SMI (women/men) (cm^2^/m^2^)	N/A	39.9 (35.8–42.7) /41.1 (39.3–42.2)	26.9–50.2 /27.4–50.2
SMI (muscle atrophy/non-muscle atrophy)	N/A	26.3%/73.7% (5/14)	N/A
PMI (women/men) (cm^2^/m^2^)	N/A	3.6 (3.4–4.6) /5.0 (4.9–5.6)	3.25–6.11 /3.48–6.07
Presence of sarcopenia (Yes/No)	N/A	31.6%/68.4% (6/13)	N/A
CS-30 (times/30 s)	N/A	10.0 (8.0–12.0)	7–22
Child-Pugh score (points)	N/A	5 (5–6)	5–12
Child-Pugh class (A/B/C)	N/A	78.9%/15.8%/5.3% (15/3/1)	N/A
Anti-diabetic medication (Yes/No)	N/A	26.3%/73.7% (5/14)	N/A
BCAA supplementation (Yes/No)	N/A	31.6%/68.4% 6/13	N/A
Biochemical examinations			
Platelet count (× 10^4^/μL)	13.3–36.9	11.0 (7.4–17.6)	4.8–26.7
AST (U/L)	10–40	33.0 (29.0–52.0)	16.0–79.0
ALT (U/L)	5–40	26.0 (18.0–43.0)	13.0–70.0
Lactate dehydrogenase (U/L)	115–245	213.0 (186.0–255.0)	166.0–355.0
ALP (U/L)	115–359	377.0 (276.0–522.0)	192.0–870.0
GGT (U/L)	0–30	37.0 (23.0–69.0)	15.0–187.0
Albumin (g/dL)	4.0–5.0	3.7 (3.0–4.1)	2.6–4.6
Prothrombin activity (%)	70–130	79.2 (67.1–86.8)	55.0–102.0
Total bilirubin (mg/dL)	0.30–1.20	0.8 (0.6–0.9)	0.3–3.1
AFP (ng/mL)	0–10	13.3 (5.8–40.2)	3.5–1216.7
des-γ-carboxy prothrombin (mAU/mL)	0–39	107 (21.0–352.0)	15–618
BUN (mg/dL)	8.0–22.0	15.5 (13.5–17.8)	6.4–28.1
Creatinine (mg/dL)	0.61–1.04	0.8 (0.6–1.0)	0.5–1.36
eGFR (mL/min/1.73 m^2^)	>90.0	65.1 (55.0–82.9)	39.1–90.0
Sodium (mmol/L)	138–145	139 (138.0–141.0)	134–142
Potassium (mmol/L)	3.6–4.8	4.0 (3.7–4.2)	2.9–5.2
HbA1c (%)	4.3–5.8	5.6 (5.4–6.8)	4.5–7.8
Ammonia (μg/dL)	13–86	79.0 (52.7–134.2)	40.0–156.0

Note: Data are expressed as median (IQR), range, or number. IQR—interquartile range; N/A—not applicable; FIM—functional independence measure; HCC—hepatocellular carcinoma; HCV—hepatitis C virus; HBV—hepatitis B virus; SMI—skeletal muscle index; PMI—psoas muscle index; CS-30, 30-s chair–stand test; ALBI—albumin–bilirubin; BCAA, branched chain amino acids; AST—aspartate aminotransferase; U/L—unit/litter; ALT—alanine aminotransferase; ALP—alkaline phosphatase; GGT—gamma-glutamyl transpeptidase; AFP—alpha-fetoprotein; BUN—blood urea nitrogen; eGFR—estimated glomerular filtration rate; HbA1c—hemoglobin A1c.

**Table 3:** Changes in liver and renal functions after physical therapy.

	Before		After		
	Median (IQR)	Range (Min–Max)	Median (IQR)	Range (Min–Max)	*p*
Child-Pugh score (point)	5 (5–6)	5–12	6 (6–7)	5–10	0.112
Child-Pugh class (A/B/C)	78.9%/15.8%/5.3% (15/3/1)	N/A	63.1%/31.6%/5.3% (12/6/1)	N/A	0.180
AST (U/L)	33.0 (29.0–52.0)	16.0–79.0	37.0 (28.0–49.0)	21.0–114.0	0.7210
ALT (U/L)	26.0 (18.0–43.0)	13.0–70.0	37.0 (23.0–56.0)	15.0–99.0	0.2579
Lactate dehydrogenase (U/L)	213.0 (186.0–255.0)	166.0–355.0	198.0 (171.0–246.0)	148.0–392.0	0.3225
ALP (U/L)	377.0 (276.0–522.0)	192.0–870.0	382.0 (305.0–479.0)	158.0–511.0	0.1703
GGT (U/L)	37.0 (23.0–69.0)	15.0–187.0	53.0 (29.0–82.0)	14.0–203.0	0.4247
eGFR (mL/min/1.73 m^2^)	65.1 (52.8–84.3)	39.1–90.0	65.8 (57.9–84.6)	34.0–99.9	0.2293

Note: Data are expressed as median (IQR), range, or number. IQR—interquartile range; N/A—not applicable; AST—aspartate aminotransferase; U/L—unit/litter; ALT—alanine aminotransferase; ALP—alkaline phosphatase; GGT—gamma-glutamyl transpeptidase; eGFR—estimated glomerular filtration rate.
